# Molecular Coverage Determines Sliding Wear Behavior of *n*-Octadecylphosphonic Acid Functionalized Cu–O Coated Steel Disks against Aluminum

**DOI:** 10.3390/ma13020280

**Published:** 2020-01-08

**Authors:** Stephan Prünte, Denis Music, Velislava L. Terziyska, Christian Mitterer, Jochen M. Schneider

**Affiliations:** 1 Materials Chemistry, RWTH Aachen University, Kopernikusstraße 10, 52074 Aachen, Germany; 2 Department of Materials Science, Montanuniversität Leoben, Franz-Josef-Straße 18, Leoben 8700, Austria

**Keywords:** friction, boundary lubrication, metal forming, organic coating

## Abstract

The sliding wear behavior of Cu–O coated steel disks functionalized with *n*-octadecyl-phosphonic acids was evaluated against aluminum in ball-on-disk tribometer experiments. After 5 m of sliding the friction coefficient of the functionalized sample with maximum molecular coverage is ≤0.3 ± 0.1. Surfaces with lower coverage mitigate friction and wear as well exhibiting initially similar low friction coefficients but reveal the breakdown of lubrication for sliding distances <5 m. The length of the low friction sliding distance before breakdown scales with the coverage of *n*-octadecylphosphonic acids on the Cu–O surface. Coverage hence determines the tribological behavior of the functionalized surface against sliding aluminum. As the coverage is increased, detrimental asperity contacts between the rubbing surfaces are reduced.

## 1. Introduction

Methods of friction mediation have accompanied human civilization from water-lubricated sleds in ancient Egypt [1,2] to brass bushes in medieval tower clocks of Salisbury and Wells Cathedral that have been working for centuries [3,4]. When modern tribology research focused on the microscopic origins of friction and possible mediation mechanisms, the role of surfactants was soon discovered, which are molecules chemically attached to and thus modifying the rubbing surfaces [5,6]. This led to the development of liquid lubricants containing surfactants such as carboxylic acids and their metallic soaps [7,8] as well as even more complex zinc dithiodialkylphosphate [9,10] as additives for metal forming and combustion engine applications, respectively. Despite significant achievements in friction reduction, friction losses are assumed to amount to 20% of the world’s energy consumption [11]. Furthermore, liquid lubrication demands extra effort in modern metal production such as near-net-shape forming processes, e.g., for automotive parts [12], due to necessary surface treatments prior and subsequent to the metal forming [13,14]. Thus, avoiding liquid lubrication could increase productivity in metal forming by up to 17% [15], since pre- and post-treatment of the workpiece are no longer required due to the absence of liquid lubrication [15]. However, adhesion between tool and workpiece needs to be prevented. Particularly, this is true for forming of aluminum, where the strong interaction between workpiece and tool surface causes rapid material transfer as well as sticking increasing friction and wear observed for aluminum-steel combinations [16,17,18] as well as for coated surfaces in contact with aluminum without liquid lubrication [19,20,21]. As already emphasized in a previous study [22], functionalizing a tool steel surface with a monolayer (ML) of *n-*octadecylphosphonic acid (C18PA) helps to reduce friction and adhesion by the distal hydrocarbon ML-moieties weakly interacting with the aluminum surface, while the ML-molecules are chemisorbed on the steel surface by P–O–Fe bonds. As the number density of atoms, molecules, or bonds in a contact defines a fundamental parameter of frictional interaction [23], we study the influence of the C18PA coverage on the sliding wear behavior against Al. Several studies on the tribological behavior of functionalized surfaces [24,25] have addressed interactions between ML and a rubbing surface [26] as well as the influence of the hydrocarbon chain length on the tribological behavior [27,28,29]. Recently, carbide precipitations in steel were reported to cause inhomogeneous molecular coverage [30]. It is well known that the molecular anchoring to the surface heavily depends on the surface composition [31,32]. Besides many different metal-oxide surfaces reacting with C18PA and forming monolayers [33,34], C18PA functionalized copper oxide surfaces have already shown friction mediation in single asperity contacts [26], while the reactivity of copper surfaces with phosphonic acids was reported to be superior compared to refractory metals, stainless steel, or aluminum [33]. This might be rationalized by surface hydroxides, which are necessary to anchor C18PA by a condensation reaction [32,34]. Consequently, Cu is deposited onto the tool steel. Due to intentional atmosphere exposure, surface oxides and hydroxides are formed [35,36]. The chemically modified Cu surfaces are henceforth referred to as Cu–O. Here we explore, for the first time, the influence of a systematic variation in molecular coverage with C18PA on the tribological behavior during sliding against aluminum.

## 2. Materials and Methods

### 2.1. Synthesis

Thin Cu–O films were obtained by non-reactive direct current sputter deposition from a Cu-target on mechanically polished tool steel disks (AISI O2/EN-90MnCrV8, Ra ≤ 0.03 µm, Martin Schleiftechnik AG, Olten, Switzerland) and subsequent atmosphere exposure. Prior to deposition, the base pressure was ≤0.5 × 10^−3^ Pa. Depositions with a target power density of 2.6 W/cm^2^ in an 0.75 Pa Ar-atmosphere resulted in an approximate 20 nm thick Cu coating after a deposition time of 30 s. Thin Cu–O films were obtained after exposure of the Cu films to the atmosphere [37]. Functionalization of as-deposited films were carried out in a different vacuum chamber (base pressure ≤ 0.5 × 10^−4^ Pa) by evaporation of *n*-octadecylphosphonic acid (C18PA or C_18_H_37_PO(OH)_2_, Sikémia, Montpellier, France, 99.9% purity) at 220 °C onto unheated Cu–O films on tool steel substrates. Subsequently, during an annealing treatment at 160 °C, surplus C18PA molecules were evaporated [38]. Prior to C18PA evaporation, the substrates were plasma cleaned using an ENI 100 reactive plasma generator (ENI, Rochester, NY, USA) applying 20 W direct current asymmetrically bipolar pulses (250 kHz) to the sample holder in an 0.75 Pa O_2_-atmosphere. Three different molecular coverages of C18PA were realized on Cu–O by variation of the evaporation time (5, 36, and 45 min).

### 2.2. Surface Characterization

Functionalized Cu–O surfaces were characterized by X-ray photoelectron spectroscopy (XPS) using a Jeol JAMP 9500F (Jeol Ltd., Tokyo, Japan) equipped with a non-monochromatic Al K_α_ X-ray source (energy 1486.6 eV, Specs GmbH, Berlin, Germany). Due to sample dimension limitations of the XPS, Si coupons of approximately 8 × 8 mm^2^ were coated with identical Cu–O thin films and functionalized alongside the Cu–O coated steel disks for equivalent ML formation and molecular coverage. Powder of the C18PA reactant pressed into an indium foil was analyzed as well. X-ray photoelectron spectra were recorded utilizing 20 eV pass energy of the detector, step sizes of 0.25 eV for a survey and 0.05 eV for detailed spectra with 5 and 20 recording cycles, respectively. The energy calibration of the hemispherical analyzer was performed by using Au 4f_7/5_ (83.98 eV), Ag 3d_5/2_ (368.26 eV), and Cu 2p_3/2_ (932.67 eV) signals. Charging was compensated by the adventitious C 1s signal (285.0 eV) [39]. Spectra analysis was carried out with the CasaXPS software (Version 2.3.19, Casa Software Ltd., Teignmouth, UK) applying mixed Gaussian–Lorentz signals and Shirley backgrounds for signal deconvolution as well as sensitivity factors determined by Wagner et al. [40] for surface chemistry calculations.

### 2.3. Tribology Testing

**Figure 1:**
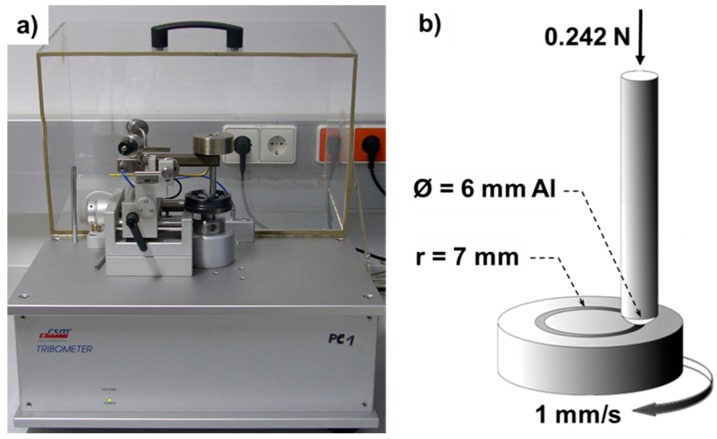
Photograph of the utilized tribometer (**a**) and a sketch of the tested pairs (**b**).

The sliding wear behavior against aluminum was investigated using a ball-on-disk test (CSM Instruments SA, Peuseux, Switzerland, Figure 1a) [16,41,42], where aluminum balls (Al 99.5) of 6 mm in diameter loaded with 0.242 N normal force were rubbed over the functionalized Cu–O coated steel disks with a sliding speed of 1 mm/s for 5 m on a 14 mm wide circular track (Figure 1b) to resemble the contact pressure in Al cold forging [22,43,44]. As great challenges in liquid lubricant-free Al-forming processes are Al-sticking and adhesion [15,19,21], ball-on-disk tests were carried out for 5 m of sliding distances to appraise the influence of molecular coverage on the tribological behavior. Each molecular coverage was tested once using a separately functionalized disk of 22 mm diameter. During ball-on-disk tests, temperature and humidity in the laboratory were kept constant at 297 K and relative humidity of 45%, respectively. Surfaces of all samples were examined after sliding tests by scanning electron microscopy (SEM) and energy dispersive X-ray analysis (EDX) with a Hitachi TM4000Plus (Hitachi, Tokyo, Japan) equipped with a backscattered electron (BSE) detector for imaging and a Bruker Quantax 75 EDX-detector (Bruker, Billerica, MA, USA). Elemental EDX-mappings with 100 measurement cycles containing 1200 × 900 pixels were quantitatively analyzed applying the QMap-toolbox of the Esprit 2.1 software (Bruker, Billerica, MA, USA) with an 8 × 8 binning. Wear track widths were determined at six equidistant and equally distributed positions utilizing a VK-9710K laser optical microscope (Keyence, Osaka, Japan) after carefully inspecting the complete wear track. Representative positions for SEM-pictures were chosen in agreement with the determined wear track width. Mass changes compared to the samples prior to ball-on-disk experiments were measured using an ABJ 80-4M electronic balance (Kern, Balingen, Germany).

## 3. Results and Discussion

### 3.1. Surface Characterization

**Figure 2:**
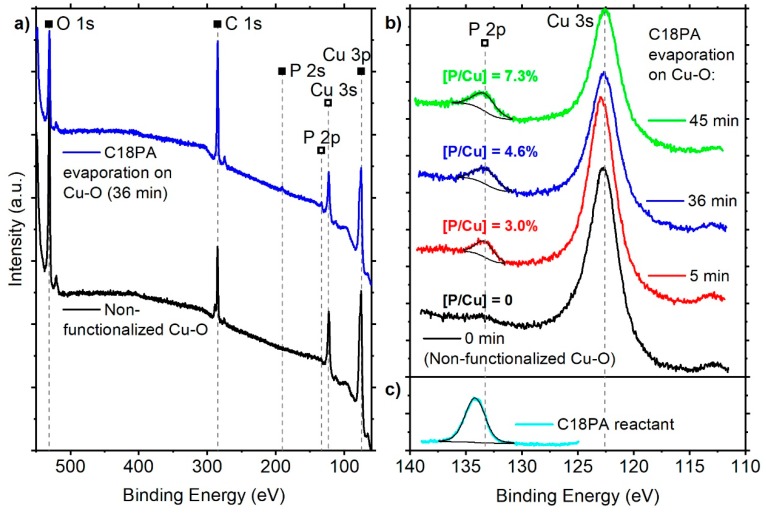
Survey (**a**) and detailed (**b**) photoelectron spectra of functionalized (colored lines) and non-functionalized (black line) Cu–O surfaces as well as of C18PA reactant (**c**) measured on indium foil. Signals labeled with filled symbols in (**a**) were used for the determination of chemical compositions in Table 1, inscriptions above P 2p signals in (**b**) refer to the P/Cu-ratio from Table 1.

X-ray photoelectron spectra were collected from all relevant surfaces as well as from powder of the C18PA reactant carefully pressed into soft indium foil. The overview spectra in Figure 2a reveal only signals stemming either from the Cu–O surface or the C18PA molecules. All surfaces with C18PA evaporation exhibit P 2p signals positioned at 133.2–133.3 eV (Figure 2b). Their full width at half-maximum (FWHM) is found at 1.8 and 2.3 eV for the 5 and 36–45 min evaporation, respectively. The P 2p signal of the C18PA reactant is located at 134.2 eV with an FWHM of 2.1 eV (Figure 2c). Evidentially, the 0.9–1.0 eV chemical shift of P 2p signals from C18PA-molecules on Cu–O surfaces compared to the non-evaporated molecules resembles the formation of P–O–M bonds as previously observed for other metal oxide surfaces [22,45,46,47]. Thus, C18PA molecules evaporated on Cu–O surfaces are chemisorbed by P–O–Cu bonds forming a monolayer with distal *n*-octadecyl residues.

The chemical composition of all surfaces was determined by utilizing the overview spectra (Figure 2a). It is evident that P is present in all functionalized surfaces (Figure 2b). The signal contribution at 133.5 eV in the non-functionalized Cu–O surface was assigned to a satellite of the Cu 3s signal [48] and no P 2s signal could be identified for this surface. Consequently, the P-concentration in the functionalized surfaces was determined by utilizing the P 2s signal due to the overlap of P 2p and Cu 3s. Evidentially, the P concentration (Table 1) in each surface varies due to different evaporation times yielding values of 1.1 at.% for 5 min C18PA evaporation and 1.5 at.% as well as 1.9 at.% for the 36 and 45 min C18PA evaporation, respectively. As the molecular anchoring consists of one P-atom per *n*-octadecyl chain bond to Cu–O by bridging O (see graphical abstract), it is reasonable to assume that the P/Cu-ratios (3.0%, 4.6%, and 7.3%) scale with the molecular coverage of C18PA. Henceforth, the three functionalized samples might be perceived as Cu–O surfaces with a relative minimal ([P/Cu] = 3.0%), medium (4.6%), and maximum (7.3%) molecular coverage of the C18PA-ML by 5, 36, and 45 min evaporation, respectively.

### 3.2. Tribology

**Figure 3:**
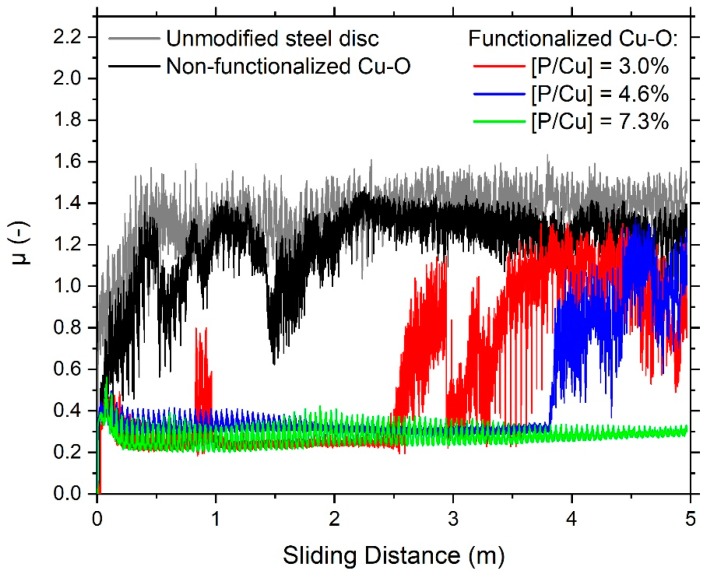
Friction coefficient µ measured for aluminum sliding over Cu–O coated steel surfaces without and with C18PA functionalization of different molecular coverages and over an unmodified steel disk. Coverages are indicated by P/Cu-ratios.

Coefficients of friction were measured for all functionalized surfaces and compared to the non-functionalized Cu–O surfaces as well as to the unmodified steel using a ball-on-disk tribometer by applying 0.242 N on an aluminum ball (Al 99.5) of 6 mm in diameter. The results reveal distinct divergences between the non-functionalized samples and the surfaces with varying C18PA-ML coverage as well as between them (Figure 3). As expected, the non-functionalized Cu–O surface and the unmodified steel disk exhibit high friction shortly after running in with friction coefficients of 0.8 to 1.4 and 1.0 to almost 1.6, respectively. Compared to the metallic surfaces, C18PA-ML on Cu–O lubricates sliding against the Al-surface revealing low friction coefficients (0.2–0.4) for at least 2.5 m of sliding, besides a short period of high friction on the C18PA-ML with minimal molecular coverage between 0.8–1.0 m. However, the low friction regime ends after 2.5 and 3.9 m of sliding for the C18PA-ML on Cu–O with minimal and medium coverage, respectively. Conversely, the sliding of aluminum against the C18PA-ML on Cu–O with the maximum coverage results in low friction for the whole sliding distance of 5 m (Figure 3).

Evidently, the functionalization of Cu–O surfaces with C18PA resulting in the formation of P–O–Cu bonds as determined by XPS (Figure 2) causes a lubricating effect reducing friction (Figure 3) due to van der Waals interactions between aluminum and the distal alkyl chains [22,26]. With coefficients of friction between 0.2 and 0.4, this lubrication is similar for all C18PA-ML on Cu–O in the low friction regime independent of molecular coverage (Figure 3). Yet, increasing the molecular coverage by longer evaporation times extends the resistance of the functionalized surface against breakdown of the low friction regime (Figure 3): The C18PA-ML with medium coverage compromises of a nearly 50% larger coverage indicated by the P-concentration (Table 1) compared to the functionalized surface with minimal coverage extending the low friction regime from 2.5 m sliding distance to 3.9 m, while the C18PA-ML with a nearly 2-fold larger coverage maintains low friction for the total sliding distance of 5 m.

To analyze the causality between molecular coverage and tribological behavior, mass changes and wear patterns were evaluated for all samples. It is evident that with increasing molecular coverage of C18PA on the Cu–O surface, mass losses as well as wear track widths were reduced (Table 2). For the sample with maximum molecular coverage of C18PA, no mass loss could be measured.

**Figure 4:**
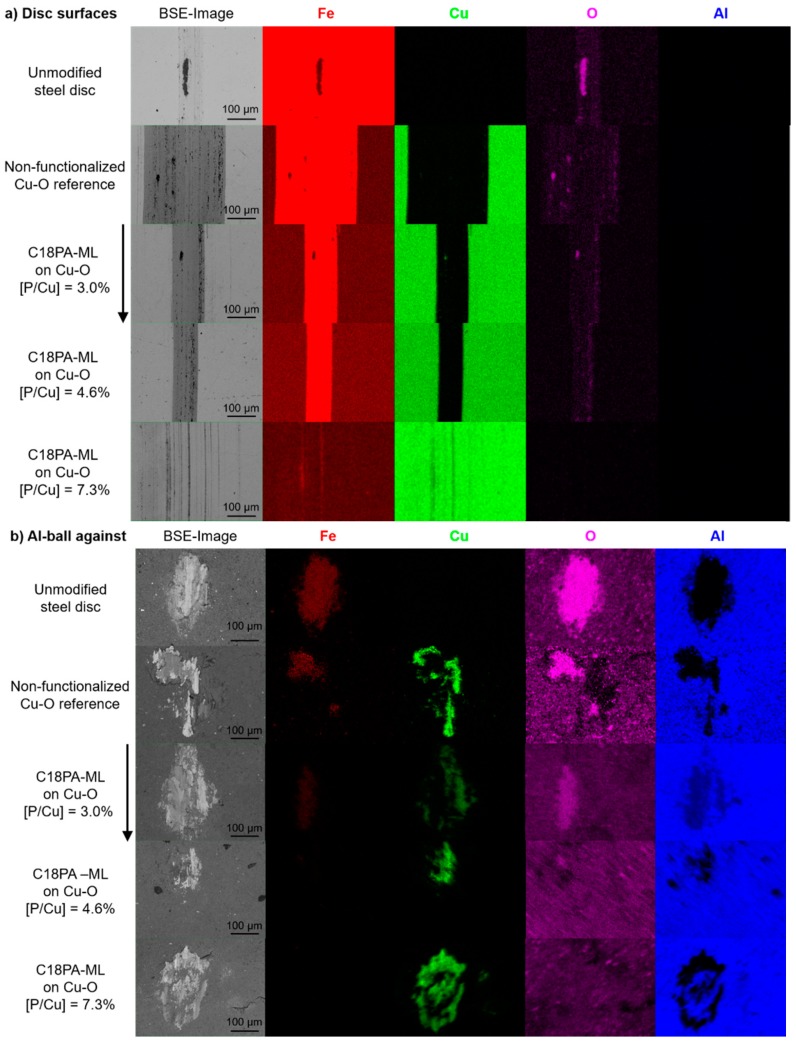
Elemental distribution of Cu–O surfaces coated on tool steel disks without and with C18PA functionalization of different molecular coverages and of an unmodified disk (**a**) as well as of corresponding aluminum counter-bodies (**b**) obtained by X-ray analysis (EDX) after ball-on-disk experiments. Coverages are indicated by the P/Cu-ratio, the sliding direction by an arrow.

Furthermore, all disks were analyzed with respect to morphology and composition by SEM and EDX, respectively (Figure 4, Table 3). The observed decrease in mass loss is reflected in the width of the wear track which was reduced as the molecular coverage increased (Figure 4a). Finally, the Cu–O coated disk with the maximum molecular coverage of C18PA does not reveal a continuous wear track after the same sliding distance. Only scratches on the surface separated by 10–20 µm can be seen (Figure 4a). Furthermore, no aluminum could be identified adhering to the disk surfaces in the ball-on-disk experiments (Figure 4a). Conversely, Cu and Fe are observed on the counter surface (Figure 4b), which is indicative of material transfer from coated and uncoated steel disks. No wear debris was observed. The size of the regions containing Cu and Fe transferred to the aluminum ball matches with the wear track width of the corresponding disk.

Evidently, the maximum molecular coverage of C18PA obstructed the formation of a wear track providing enhanced tribological stability (Figure 4a). However, wear tracks of functionalized Cu–O with minimal and medium coverage are significantly smaller than on the non-functionalized disk possibly due to adhesive failure of Cu–O. Yet, sliding against aluminum revealed immediately high friction (Figure 3) and material transfer (Figure 4b) for the unmodified steel disk similar to previous studies [16,17,18] as well as for the disk with the non-functionalized Cu–O surface baring the widest wear track.

The functionalization of Cu–O with C18PA of maximum molecular coverage outperforms all other surfaces considering the low friction against aluminum for the complete sliding distance (Figure 3), minor surface damages (Figure 4a), and no mass loss (Table 2). In agreement with the boundary lubrication model [8,25], the Cu–O functionalization with the maximum molecular coverage produces a protective monolayer against penetrating aluminum asperities leading to only a few distant scratches on the surface of the disk with maximized coverage (Figure 4a) and a large plastic deformation of the corresponding aluminum-ball distributing transferred material over the complete contact (Figure 4b). Hence both, the length of the low friction regime in the ball-on-disk experiments (Figure 3) and wear patterns (Table 2, Figure 4) scale with molecular coverage highlighting the here presented correlation between tribological behavior and the spatial coverage of Cu–O surfaces functionalized by C18PA. The enhanced tribological stability may be rationalized by considering that with increasing molecular coverage of chemisorbed C18PA molecules harmful asperity contacts become less likely enabling a stable coefficient of friction and minor surface damage. Consequently, the variation in molecular coverage of C18PA functionalized Cu–O surfaces influences fundamentally the sliding wear behavior against aluminum and hence the presented functionalization strategy with maximum molecular coverage may help to improve applications in aluminum forming.

## 4. Conclusions

The tribological behavior of Cu–O surfaces functionalized with monolayers of *n*-octadecyl-phosphonic acids was investigated in sliding experiments against aluminum. The molecular coverage was varied by employing different evaporation times. While all monolayers firmly attached by P–O–Cu bonds mitigate friction and wear significantly compared to a non-functionalized surface, their lifetime and breakdown of boundary lubrication are determined by their molecular coverage. We attribute this behavior to the prevention of detrimental asperity contacts averted by the maximized molecular coverage, which causes a narrow spatial arrangement of the distal *n*-octadecyl hydrocarbon chains. The preparation of monolayers by vacuum thermal evaporation and a subsequent functionalization by annealing is ideally suited to vary the molecular coverage of C18PA on Cu–O. As this research strategy is not confined to a particular functionalization agent, it enables the investigation of coverage induced changes on tribological behavior in environmental-friendly forming applications.

## Figures and Tables

**Table 1:** Chemical composition of C18PA reactant as well as of functionalized and non-functionalized surfaces obtained by photoelectron signals of elements labeled with filled symbols in Figure 2a.

Sample	In (at.%)	O (at.%)	C (at.%)	P (at.%)	Cu (at.%)	(P/Cu) (%)
C18PA reactant on indium foil	0.6	16.8	78.9	3.8	–	–
Non-functionalized Cu–O	–	34.2	27.6	–	38.2	–
5 min C18PA evaporation	–	30.1	31.6	1.1	37.1	3.0
36 min C18PA evaporation	–	18.1	47.6	1.5	32.8	4.6
45 min C18PA evaporation	–	23.3	49.1	1.9	25.7	7.3

**Table 2:** Mass change (Δm) compared to sample mass prior to ball-on-disk experiments and wear track width of tool steel.

Sample	Δm (g)	Wear Track Width (µm)
Unmodified steel disk	0.00 ± 0.01	95 ± 5
Non-functionalized	−0.07 ± 0.01	281 ± 1
[P/Cu] = 3.0%	−0.03 ± 0.01	125 ± 5
[P/Cu] = 4.6%	−0.02 ± 0.01	84 ± 3
[P/Cu] = 7.3%	0.00 ± 0.01	No track established

**Table 3:** Chemical composition of the tested pairs as shown in Figure 4 determined by EDX.

Disk Surface	Fe (at. %)	Cu (at. %)	O (at. %)	Al (at. %)
Unmodified steel disk	90.6	–	9.4	–
Non-functionalized Cu–O reference	54.2	38.1	7.7	–
[P/Cu] = 3.0%	39.4	55.0	5.6	–
[P/Cu] = 4.6%	42.3	52.2	5.5	–
[P/Cu] = 7.3%	25.5	72.4	2.1	–
**Al-ball against**				
Unmodified steel disk	4.3	-	30.7	65.0
Non-functionalized Cu–O reference	1.9	4.0	27.2	66.9
[P/Cu] = 3.0%	1.0	2.4	22.0	74.6
[P/Cu] = 4.6%	0.4	1.7	26.6	71.3
[P/Cu] = 7.3%	0.0	6.2	19.0	74.8
